# Supplementary material for: Multi-step coordination of telomerase recruitment in fission yeast through two coupled telomere-telomerase interfaces
Source: eLife. 2016 Jun 2;5:e15470. doi: 10.7554/eLife.15470 (PMC4936895; doi:10.7554/eLife.15470)
Supplement: Supplementary file 1. — DOI: http://dx.doi.org/10.7554/eLife.15470.015 [file elife-15470-supp1.docx]

| **Supplemental Table S1.** Fission yeast strains used in this study | | |
| --- | --- | --- |
| ***Figure*** | ***Strain*** | ***Full genotype*** |
| 1B | FQ29 | *h^+^ ade6-M216 leu1-32 ura4-D18 his3-D1* |
|  | JL99 | *h^+^ ade6-M216 leu1-32 ura4-D18 his3-D1 tpz1-E74R-5Flag-kanMx6* |
|  | XH164 | *h^+^ ade6-M216 leu1-32 ura4-D18 his3-D1 tpz1-K75E-5Flag-kanMx6* |
|  | JL101 | *h^+^ ade6-M216 leu1-32 ura4-D18 his3-D1 tpz1-R76E-5Flag-kanMx6* |
|  | JL102 | *h^+^ ade6-M216 leu1-32 ura4-D18 his3-D1 tpz1-I77R-5Flag-kanMx6* |
|  | JL103 | *h^+^ ade6-M216 leu1-32 ura4-D18 his3-D1 tpz1-R81E-5Flag-kanMx6* |
|  |  |  |
| 1C | FQ29 | *h^+^ ade6-M216 leu1-32 ura4-D18 his3-D1* |
|  | XH248 | *h^-^ ade6-M216 or M210 leu1-32 ura4-D18 his3-D1 trt1::his3* |
|  | XH247 | *h^+^ade6-M216 or M210 leu1-32 ura4-D18 his3-D1 tpz1-R81E-5Flag-kanMx6/trt1::his3* |
|  |  |  |
| 1D | FQ29 | *h^+^ ade6-M216 leu1-32 ura4-D18 his3-D1* |
|  | XH198 | *h^-^ ade6-M216 or WT leu1-32 ura4-D18 his3-D1 or WT tpz1-5Flag-kanMx6/poz1::hyg* |
|  | XH201 | *h^+^ade6-M216 or WT leu1-32 ura4-D18 his3-D1 or WT tpz1-R81E-5Flag-kanMx6/poz1::hyg* |
|  |  |  |
| 2A | HTJ1 | *h^+^ ade6-M216 leu1-32 ura4-D18 his3-D1 tpz1-wt-5Flag-kanMX6* |
|  | JL413 | *h^-^ ade6-M210 trt1-9PK-kanMx6* |
|  | JL433 | *h^-^ ade6-M216 or M210 leu1-32 or WT ura4-D18 or WT his3-D1 or WT trt1-9PK-kanMx6/tpz1-5Flag-kanMx6* |
|  | JL434 | *h^-^ ade6-M216 or M210 leu1-32 or WT ura4-D18 or WT his3-D1 or WT trt1-9PK-kanMx6/tpz1-E74R-5Flag-kanMx6* |
|  | JL435 | *h^-^ ade6-M216 or M210 leu1-32 or WT ura4-D18 or WT his3-D1 or WT trt1-9PK-kanMx6/tpz1-R76E-5Flag-kanMx6* |
|  |  |  |
| 2B | JL413 | *h^-^ ade6-M210 trt1-9PK-kanMx6* |
|  | JL433 | *h^-^ ade6-M216 or M210 leu1-32 or WT ura4-D18 or WT his3-D1 or WT trt1-9PK-kanMx6/tpz1-5Flag-kanMx6* |
|  | JL436 | *h^-^ ade6-M216 or M210 leu1-32 or WT ura4-D18 or WT his3-D1 or WT trt1-9PK-kanMx6/tpz1-I77R-5Flag-kanMx6* |
|  | JL437 | *h^-^ ade6-M216 or M210 leu1-32 or WT ura4-D18 or WT his3-D1 or WT trt1-9PK-kanMx6/tpz1-R81E-5Flag-kanMx6* |
|  |  |  |
| 2E | HTJ1 | *h^+^ ade6-M216 leu1-32 ura4-D18 his3-D1 tpz1-wt-5Flag-kanMX6* |
|  | JL413 | *h^-^ ade6-M210 trt1-9PK-kanMx6* |
|  | JL433 | *h^-^ ade6-M216 or M210 leu1-32 or WT ura4-D18 or WT his3-D1 or WT trt1-9PK-kanMx6/tpz1-5Flag-kanMx6* |
|  | JL434 | *h^-^ ade6-M216 or M210 leu1-32 or WT ura4-D18 or WT his3-D1 or WT trt1-9PK-kanMx6/tpz1-E74R-5Flag-kanMx6* |
|  | JL435 | *h^-^ ade6-M216 or M210 leu1-32 or WT ura4-D18 or WT his3-D1 or WT trt1-9PK-kanMx6/tpz1-R76E-5Flag-kanMx6* |
|  | JL436 | *h^-^ ade6-M216 or M210 leu1-32 or WT ura4-D18 or WT his3-D1 or WT trt1-9PK-kanMx6/tpz1-I77R-5Flag-kanMx6* |
|  | JL437 | *h^-^ ade6-M216 or M210 leu1-32 or WT ura4-D18 or WT his3-D1 or WT trt1-9PK-kanMx6/tpz1-R81E-5Flag-kanMx6* |
|  |  |  |
| 2F | JL413 | *h^-^ ade6-M210 trt1-9PK-kanMx6* |
|  | JL433 | *h^-^ ade6-M216 or M210 leu1-32 or WT ura4-D18 or WT his3-D1 or WT trt1-9PK-kanMx6/tpz1-5Flag-kanMx6* |
|  | XH391 | *h^-^ ade6-M216 or M210 leu1-32 or WT ura4-D18 or WT his3-D1 or WT tpz-5Flag-kanMx6/trt1-9PK-kanMx6/ccq1-T93A-stop-13myc-hphMx6* |
|  | XH421 | *h^-^ ade6-M216 or M210 leu1-32 or WT ura4-D18 his3-D1 ccq1-13myc-hphMx6/tpz1-5Flag-kanMx6/trt1-9PK-kanMx6* |
|  | XH424 | *h^+^ ade6-M216 or M210 leu1-32 or WT ura4-D18 or WT his3-D1 or WT ccq1-F157A/K174E-13myc-hphMx6/tpz1-5Flag-kanMx6/trt1-9PK-kanMx6* |
|  |  |  |
| 3A | FQ29 | *h^+^ ade6-M216 leu1-32 ura4-D18 his3-D1* |
|  | FQ37 | *h^-^ leu1-32 ura4-D18 his3-D1 trt1-G8-13myc-kanMx6* |
|  | HIJ104 | *h^-^ ade6-M216 ura4-D18 his3-D1 trt1-G8-13myc-kanMx6/ccq1-T93A-5Flag-kanMx6* |
|  | JL136 | *h^-^ ade6-M216 leu1-32 ura4-D18 his3-D1 trt1-G8-13myc-kanMx6/tpz1-E74R-5Flag-kanMx6* |
|  | JL137 | *h^-^ ade6-M216 leu1-32 ura4-D18 his3-D1 trt1-G8-13myc-kanMx6/tpz1-R76E-5Flag-kanMx6* |
|  | JL138 | *h^-^ ade6-M216 leu1-32 ura4-D18 his3-D1 trt1-G8-13myc-kanMx6/tpz1-I77R-5Flag-kanMx6* |
|  | JL139 | *h^-^ ade6-M216 leu1-32 ura4-D18 his3-D1 trt1-G8-13myc-kanMx6/tpz1-R81E-5Flag-kanMx6* |
|  |  |  |
| 3B | FQ51 | *h^-^ leu1-32 ura4-D18 ccq1::hyg* |
|  | HTJ1 | *h^+^ ade6-M216 leu1-32 ura4-D18 his3-D1 tpz1-wt-5Flag-kanMX6* |
|  | JL99 | *h^+^ ade6-M216 leu1-32 ura4-D18 his3-D1 tpz1-E74R-5Flag-kanMx6* |
|  | XH164 | *h^+^ ade6-M216 leu1-32 ura4-D18 his3-D1 tpz1-K75E-5Flag-kanMx6* |
|  | JL101 | *h^+^ ade6-M216 leu1-32 ura4-D18 his3-D1 tpz1-R76E-5Flag-kanMx6* |
|  | JL102 | *h^+^ ade6-M216 leu1-32 ura4-D18 his3-D1 tpz1-I77R-5Flag-kanMx6* |
|  | JL103 | *h^+^ ade6-M216 leu1-32 ura4-D18 his3-D1 tpz1-R81E-5Flag-kanMx6* |
|  |  |  |
| 3C | FQ29 | *h^+^ ade6-M216 leu1-32 ura4-D18 his3-D1* |
|  | HTJ1 | *h^+^ ade6-M216 leu1-32 ura4-D18 his3-D1 tpz1-wt-5Flag-kanMX6* |
|  | JL99 | *h^+^ ade6-M216 leu1-32 ura4-D18 his3-D1 tpz1-E74R-5Flag-kanMx6* |
|  | XH164 | *h^+^ ade6-M216 leu1-32 ura4-D18 his3-D1 tpz1-K75E-5Flag-kanMx6* |
|  | JL101 | *h^+^ ade6-M216 leu1-32 ura4-D18 his3-D1 tpz1-R76E-5Flag-kanMx6* |
|  | JL102 | *h^+^ ade6-M216 leu1-32 ura4-D18 his3-D1 tpz1-I77R-5Flag-kanMx6* |
|  | JL103 | *h^+^ ade6-M216 leu1-32 ura4-D18 his3-D1 tpz1-R81E-5Flag-kanMx6* |
|  |  |  |
| 4A | FQ29 | *h^+^ ade6-M216 leu1-32 ura4-D18 his3-D1* |
|  | JL416 | *h^+^/h^-^ ade6-M216/M210 tpz1::kanMX6/+ trt1-9PK-tpz1-natMX6/+* |
|  | XH155 | *h^+^ ade6-M216 or M210 trt1-9PK-tpz1-natMx6/tpz1::kanMx6* |
|  | XH116 | *h^+^ ade6-M216 or M210 trt1-9PK-tpz1-E74R-natMx6/tpz1::kanMx6* |
|  | XH111 | *h^+^ ade6-M216 or M210 trt1-9PK-tpz1-R76E-natMx6/tpz1::kanMx6* |
|  | XH115 | *h^+^ ade6-M216 or M210 trt1-9PK-tpz1-I77R-natMx6/tpz1::kanMx6* |
|  | XH112 | *h^+^ ade6-M216 or M210 trt1-9PK-tpz1-R81E-natMx6/tpz1::kanMx6* |
|  | JL452 | *h^+^ ade6-M216 or M210 trt1-9PK-tpz1-L449A-natMx6/tpz1::kanMx6* |
|  |  |  |
| 4B | FQ29 | *h^+^ ade6-M216 leu1-32 ura4-D18 his3-D1* |
|  | JL413 | *h^-^ ade6-M210 trt1-9PK-kanMx6* |
|  | JL416 | *h^+^/h^-^ ade6-M216/M210 tpz1::kanMX6/+ trt1-9PK-tpz1-natMX6/+* |
|  | XH155 | *h^+^ ade6-M216 or M210 trt1-9PK-tpz1-natMx6/tpz1::kanMx6* |
|  | XH116 | *h^+^ ade6-M216 or M210 trt1-9PK-tpz1-E74R-natMx6/tpz1::kanMx6* |
|  | XH111 | *h^+^ ade6-M216 or M210 trt1-9PK-tpz1-R76E-natMx6/tpz1::kanMx6* |
|  | XH115 | *h^+^ ade6-M216 or M210 trt1-9PK-tpz1-I77R-natMx6/tpz1::kanMx6* |
|  | XH112 | *h^+^ ade6-M216 or M210 trt1-9PK-tpz1-R81E-natMx6/tpz1::kanMx6* |
|  | JL452 | *h^+^ ade6-M216 or M210 trt1-9PK-tpz1-L449A-natMx6/tpz1::kanMx6* |
|  |  |  |
| 5A | XH37 | *h^+^ ade6-M216 leu1-32 ura4-D18 his3-D1 //pREP41-2Myc-est1* |
|  | XH249 | *h^-^ ade6-M216 ura4-D18 his3-D1 ccq1-5Flag-kanMx6 //pREP41-2Myc-est1* |
|  | XH250 | *h^-^ ade6-M216 ura4-D18 his3-D1 ccq1-T93A-5Flag-kanMx6 //pREP41-2Myc-est1* |
|  | XH366 | *h^-^ ade6-M210 leu1-32 ura4-D18 his3-D1 ccq1-V152R-5Flag-kanMx6 //pREP41-2Myc-est1* |
|  | XH323 | *h^-^ ade6-M210 leu1-32 ura4-D18 his3-D1 ccq1-F157A/K174E-5Flag-kanMx6 //pREP41-2Myc-est1* |
|  | XH370 | *h^-^ ade6-M210 leu1-32 ura4-D18 his3-D1 ccq1-I175R-5Flag-kanMx6 //pREP41-2Myc-est1* |
|  | XH371 | *h^-^ ade6-M210 leu1-32 ura4-D18 his3-D1 ccq1-L177R-5Flag-kanMx6 //pREP41-2Myc-est1* |
|  |  |  |
| 5C | JL397 | *h^-^ leu1-32 ura4-D18 his3-D1 est1-13myc-hphMx6* |
|  | JL402 | *h^-^ leu1-32 ura4-D18 his3-D1 ccq1-5Flag-kanMx6/est1-13myc-hphMx6* |
|  | JL403 | *h^-^ leu1-32 ura4-D18 his3-D1 ccq1-T93A-5Flag-kanMx6/est1-13myc-hphMx6* |
|  | XH443 | *h^-^ leu1-32 ura4-D18 his3-D1 ccq1-T93A/F157A/K174E-5Flag-kanMx6/est1-13myc-hphMx6* |
|  | XH444 | *h^+^ ade6-M216 or M210 leu1-32 or WT ura4-D18 or WT his3-D1 or WT ccq1-T93A-5Flag-kanMx6/est1-K252E-G8-13myc-kanMx6* |
|  | XH446 | *h^+^ ade6-M216 or 210 leu1-32 or WT ura4-D18 his3-D1 ccq1-T93A-5Flag-kanMx6/est1-R79A/R180A-13myc-kanMx6* |
|  | XH445 | *h^-^ ade6-M210 leu1-32 ura4-D18 or WT his3-D1 or WT ccq1-F157A/K174E-5Flag-kanMx6/est1-K252E-G8-13myc-kanMx6* |
|  |  |  |
| 5E | FQ29 | *h^+^ ade6-M216 leu1-32 ura4-D18 his3-D1* |
|  | XH37 | *h^+^ ade6-M216 leu1-32 ura4-D18 his3-D1 //pREP41-2Myc-est1* |
|  | XH249 | *h^-^ ade6-M216 ura4-D18 his3-D1 ccq1-5Flag-kanMx6 //pREP41-2Myc-est1* |
|  |  |  |
| 6A | XH451 | *h^+^ ade6-M216 or WT leu1-32 ura4-D18 his3-D1 cdc25-22* |
|  | XH460 | *h^-^ ade6-M216 or M210 or WT leu1-32 or WT ura4-D18 or WT his3-D1 or WT tpz1-5Flag-kanMx6/trt1-9PK-kanMx6/cdc25-22* |
|  | XH391 | *h^-^ ade6-M216 or M210 leu1-32 or WT ura4-D18 or WT his3-D1 or WT tpz-5Flag-kanMx6/trt1-9PK-kanMx6/ccq1-T93A-stop-13myc-hphMx6* |
|  |  |  |
| 6B | XH451 | *h^+^ ade6-M216 or WT leu1-32 ura4-D18 his3-D1 cdc25-22* |
|  | XH473 | *h^-^ ade6-M216 or M210 or WT leu1-32 ura4-D18 or WT his3-D1 or WT tpz1-5Flag-kanMx6/trt1-9PK-kanMx6/ccq1-T93A-stop-13myc-hphMx6/cdc25-22* |
|  | JL433 | *h^-^ ade6-M216 or M210 leu1-32 or WT ura4-D18 or WT his3-D1 or WT trt1-9PK-kanMx6/tpz1-5Flag-kanMx6* |
|  |  |  |
| 6E | XH451 | *h^+^ ade6-M216 or WT leu1-32 ura4-D18 his3-D1 cdc25-22* |
|  | XH459 | h*^+^ ade6-M216 or WT leu1-32 ura4-D18 his3-D1 est1-13myc-hphMx6/cdc25-22* |
|  | JL403 | *h^-^ leu1-32 ura4-D18 his3-D1 ccq1-T93A-5Flag-kanMx6/est1-13myc-hphMx6* |
|  |  |  |
| 6F | XH451 | *h^+^ ade6-M216 or WT leu1-32 ura4-D18 his3-D1 cdc25-22* |
|  | XH481 | h*^-^ ade6-M216 or WT leu1-32 ura4-D18 his3-D1 est1-13myc-hphMx6/ccq1-T93A-5Flag-kanMx6/cdc25-22* |
|  | JL402 | *h^-^ leu1-32 ura4-D18 his3-D1 ccq1-5Flag-kanMx6/est1-13myc-hphMx6* |
|  |  |  |
| 1-S1 | FQ29 | *h^+^ ade6-M216 leu1-32 ura4-D18 his3-D1* |
|  | HIJ107 | *h^-^ ade6-M216 ura4-D18 his3-D1 ccq1-T93A-5Flag-kanMx6* |
|  | JL98 | *h^+^ ade6-M216 leu1-32 ura4-D18 his3-D1 tpz1-T73A-5Flag-kanMx6* |
|  | JL99 | *h^+^ ade6-M216 leu1-32 ura4-D18 his3-D1 tpz1-E74R-5Flag-kanMx6* |
|  | JL101 | *h^+^ ade6-M216 leu1-32 ura4-D18 his3-D1 tpz1-R76E-5Flag-kanMx6* |
|  | JL104 | *h^+^ ade6-M216 leu1-32 ura4-D18 his3-D1 tpz1-I105R-5Flag-kanMx6* |
|  | JL105 | *h^+^ ade6-M216 leu1-32 ura4-D18 his3-D1 tpz1-V107R-5Flag-kanMx6* |
|  | JL106 | *h^+^ ade6-M216 leu1-32 ura4-D18 his3-D1 tpz1-E108R-5Flag-kanMx6* |
|  | JL107 | *h^+^ ade6-M216 leu1-32 ura4-D18 his3-D1 tpz1-F112A-5Flag-kanMx6* |
|  | JL108 | *h^+^ ade6-M216 leu1-32 ura4-D18 his3-D1 tpz1-E116R-5Flag-kanMx6* |
|  | JL109 | *h^+^ ade6-M216 leu1-32 ura4-D18 his3-D1 tpz1-K124E-5Flag-kanMx6* |
|  |  |  |
| 1-S2 | FQ29 | *h^+^ ade6-M216 leu1-32 ura4-D18 his3-D1* |
|  | HTJ1 | *h^+^ ade6-M216 leu1-32 ura4-D18 his3-D1 tpz1-wt-5Flag-kanMX6* |
|  | JL98 | *h^+^ ade6-M216 leu1-32 ura4-D18 his3-D1 tpz1-T73A-5Flag-kanMx6* |
|  | JL99 | *h^+^ ade6-M216 leu1-32 ura4-D18 his3-D1 tpz1-E74R-5Flag-kanMx6* |
|  | XH164 | *h^+^ ade6-M216 leu1-32 ura4-D18 his3-D1 tpz1-K75E-5Flag-kanMx6* |
|  | JL101 | *h^+^ ade6-M216 leu1-32 ura4-D18 his3-D1 tpz1-R76E-5Flag-kanMx6* |
|  | JL102 | *h^+^ ade6-M216 leu1-32 ura4-D18 his3-D1 tpz1-I77R-5Flag-kanMx6* |
|  | JL103 | *h^+^ ade6-M216 leu1-32 ura4-D18 his3-D1 tpz1-R81E-5Flag-kanMx6* |
|  | JL104 | *h^+^ ade6-M216 leu1-32 ura4-D18 his3-D1 tpz1-I105R-5Flag-kanMx6* |
|  | JL105 | *h^+^ ade6-M216 leu1-32 ura4-D18 his3-D1 tpz1-V107R-5Flag-kanMx6* |
|  | JL106 | *h^+^ ade6-M216 leu1-32 ura4-D18 his3-D1 tpz1-E108R-5Flag-kanMx6* |
|  | JL107 | *h^+^ ade6-M216 leu1-32 ura4-D18 his3-D1 tpz1-F112A-5Flag-kanMx6* |
|  | JL108 | *h^+^ ade6-M216 leu1-32 ura4-D18 his3-D1 tpz1-E116R-5Flag-kanMx6* |
|  | JL109 | *h^+^ ade6-M216 leu1-32 ura4-D18 his3-D1 tpz1-K124E-5Flag-kanMx6* |
|  |  |  |
| 1-S3 | FQ29 | *h^+^ ade6-M216 leu1-32 ura4-D18 his3-D1* |
|  | XH201 | *h^+^ade6-M216 or WT leu1-32 ura4-D18 his3-D1 or WT tpz1-R81E-5Flag-kanMx6/poz1::hyg* |
|  |  |  |
| 2-S1 | FQ29 | *h^+^ ade6-M216 leu1-32 ura4-D18 his3-D1* |
|  | JL413 | *h^-^ ade6-M210 trt1-9PK-kanMx6* |
|  | XH197 | *h^-^ ade6-M216 leu1-32 or WT ura4-D18 or WT his3-D1 or WT tpz1-K75E-5Flag-kanMx6/trt1-9PK-kanMx6* |
|  | JL438 | *h^-^ ade6-M210 or M216 leu1-32 or WT ura4-D18 or WT his3-D1 or WT tpz1-K75A-5Flag-kanMx6/trt1-9PK-kanMx6* |
